# Smart Inlays for Simultaneous Crack Sensing and Arrest in Multifunctional Bondlines of Composites

**DOI:** 10.3390/s21113852

**Published:** 2021-06-02

**Authors:** Chresten von der Heide, Julian Steinmetz, Martin J. Schollerer, Christian Hühne, Michael Sinapius, Andreas Dietzel

**Affiliations:** 1 Institute of Microtechnology, Technische Universität Braunschweig, 38124 Braunschweig, Germany; a.dietzel@tu-braunschweig.de; 2 Institute of Mechanics and Adaptronics, Technische Universität Braunschweig, 38106 Braunschweig, Germany; j.steinmetz@tu-braunschweig.de (J.S.); or christian.huehne@dlr.de (C.H.); m.sinapius@tu-braunschweig.de (M.S.); 3 Institute of Composite Structures and Adaptive Systems, German Aerospace Center (DLR), 38108 Braunschweig, Germany; martin.schollerer@dlr.de

**Keywords:** thin-film sensors, foil sensors, composite structures, structural bonding, multifunctional bondline, function conformity, sensor integration

## Abstract

Disbond arrest features combined with a structural health monitoring system for permanent bondline surveillance have the potential to significantly increase the safety of adhesive bonds in composite structures. A core requirement is that the integration of such features is achieved without causing weakening of the bondline. We present the design of a smart inlay equipped with a micro strain sensor-system fabricated on a polyvinyliden fluorid (PVDF) foil material. This material has proven disbond arrest functionality, but has not before been used as a substrate in lithographic micro sensor fabrication. Only with special pretreatment can it meet the requirements of thin film sensor elements regarding surface roughness and adhesion. Moreover, the sensor integration into composite material using a standard manufacturing procedure reveals that the smart inlays endure this process even though subjected to high temperatures, curing reactions and plasma treatment. Most critical is the substrate melting during curing when sensory function is preserved with a covering caul plate that stabilizes the fragile measuring grids. The smart inlays are tested by static mechanical loading, showing that they can be stretched far beyond critical elongations of composites before failure. The health monitoring function is verified by testing the specimens with integrated sensors in a cantilever bending setup. The results prove the feasibility of micro sensors detecting strain gradients on a disbond arresting substrate to form a so-called multifunctional bondline.

## 1. Introduction

Fiber reinforced plastics (FRPs) offer an abundance of applications with the benefits of structural improvements at high mechanical load and low weight [1,2]. Structural health monitoring (SHM) of advanced materials, such as FRP by integrated sensors, is key to fully exploiting their potential. While joining technologies for conventional isotropic materials such as metal are well established, most composite fabrication processes are complex and safely joining the individual FRP components is still demanding [1]. Adhesive bonds promise the best suited technology for light-weight FRP due to their planar load transfer and their low weight in comparison to any form of bolted joint. However, they are prone to environmental degradation and the emergence of some severe defects is not detectable [3,4]. This keeps industry from the broad implementation of structural, i.e., high load bearing adhesive bonds, for safety critical parts [5]. A smart system for permanent adhesive layer surveillance with crack suppressing properties that reliably prevents catastrophic failure could thus be a breakthrough.

Flexible foil-based sensor systems are ideally suited for integration or application to FRP materials [6]. Next to polyimide (PI) as a substrate material, which can be preferably processed in a liquid form for subsequent sensor microfabrication [7,8,9], other substrate materials like PEN and PET foil materials have been used for flexible electronics fabrication [10]. An overview of previous substrate choices and fabrication concepts has already been provided [11], where the importance of balancing material properties like thermal expansion, elastic moduli and adhesion strength for reliable operation of systems-in-foil under mechanical bending and at varied temperatures is outlined. Flexible sensor elements can be supplemented by active microelectronic components made of thin silicon without decisively limiting the flexibility [12]. Moreover, rigid sensors can also be combined with flexible sensors, as shown by the example of a pressure sensor that allows flip-chip assembly in foil-based flexible systems to be used in harsh environments [13].

A common way to monitor load conditions is the use of commercial strain gages glued to the components surface. For the highly stressable FRP materials, special high-performance types exist to withstand large dynamic loads and strains. For such sensors, the foil substrate is decisive for its long-term durability. PI is a common sensor substrate, due to its high mechanical load capacity, chemical stability and excellent electrical properties. For FRP applications with many load cycles, however, even more stable substrate materials such as glass fiber reinforced phenolic resin are used, in order to avoid critical strain transferred to the metallic measuring grid.

If internal strains shall be monitored, the external application of sensors is not sufficient. Due to the layer structure of FRP materials, interlaminar sensor integration can be achieved with some specially designed (foil) sensors, fiber Bragg grating (FBG) or standard strain gages, whose electrical contacting is made possible by means of vertical externally insulated pins piercing the laminate layers. However, while externally applied sensors typically exhibit no influence on the mechanical properties of the structural components, the load-bearing capacity of the latter systems is often reduced due to interlaminar integrated sensors [14,15,16]. Possible internal stress peaks resulting from the sensor integration can cause damages such as cracks and delaminations [2]. Thus, the interlaminar integration of such sensors as well as other sensor variants is in the focus of current research.

Until now, this research mainly focused on minimizing the undesired side effects of sensor embedding. Thus, a list that includes the six most important challenges of sensor integration was created, including the pursuit of designing a small volume, flexible sensor that has the required long-term stability [2]. Numerical simulations show that the interference from sensors integrated into FRPs is lowest if they are embedded in the form of flexible foil-like inlays [15]. With a foil sensor based on a 10 μm thin PI substrate with holes for the cure monitoring of composites, those numeric results were confirmed. Here, no significant reduction in the strength of the composite by the integrated foil sensor in three point bending and interlaminar shear tests was reported [1]. However, PI substrates have been shown to possess weak adhesion to epoxy, which promotes internal delaminations. However, it was demonstrated that polyetherimide (PEI) has no such tendency and very good adhesion to epoxy [17]. In addition, the sensor embedding process into compound materials is a major challenge, as high temperatures and mechanical stress must be endured and chemical resistance is inevitable [18,19,20,21].

In SHM systems especially designed for adhesive layer monitoring, the sensors exploit a variety of detection techniques [22,23,24,25]. Strain measurements within the adhesive layer using FBG sensors are an increasingly common method. Various experiments have proven the applicability of the FBG technology [26,27]. Filigree sensors can either be embedded into the composite matrix [28,29,30] or directly into the adhesive layer [31,32]. There is even evidence that the integration of particularly thin FBG sensors can diminish the effect of load capacity reduction due to embedded sensors [33].

The concept of minimally invasive sensors, sensor materials adapted to the surrounding matrix and a minimized overall sensor volume, was named function scale integration [34]. The concept of combining function scale integration with crack arresting structures was already proposed under the term multifunctional disbond arrest feature (MDAF) [35]. Smart polymer inlays co-cured to the carbon fiber reinforced plastic (CFRP) adherends act as sensor substrate and crack arrest materials simultaneously. Crack propagation is assumed to be stopped by the 10 mm polyvinyliden fluoride (PVDF) strips in analogy to the surface toughening (ST) principle [36,37]. The idea is to reduce the stiffness near the crack front, such that the induced stress peak is flattened and load-transfer to the adhesive layer behind the inlay is improved, which in turn reduces the peeling load. With the implementation of the MDAF, sensor integration does not weaken the adhesive layer, but improves its robustness by adding further functionality. Hence, it is a promising approach to achieving function conformity, which means that every function shall be fulfilled as well as possible, while minimally disturbing the other ones [38]. The goal of the work at hand was to develop smart inlays on crack stopping PVDF foils carrying an array of micro sensors. CFRP integration of such smart inlays using conventional composite processes shall demonstrate the usability of the MDAF concept for industrial applications.

## 2. Design of the Smart Inlay Inside the Multifunctional Disbond Arrest Feature

**Figure 1:**
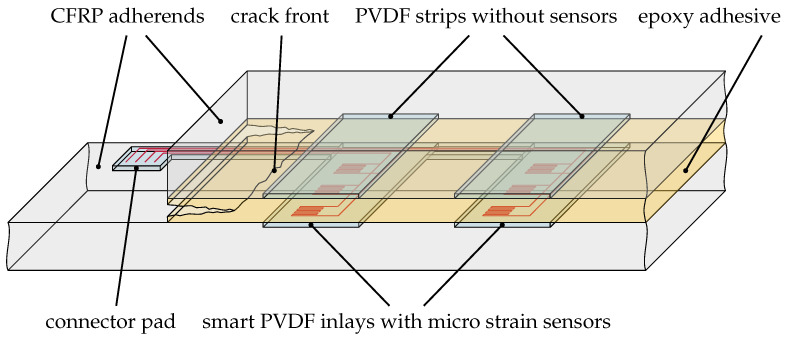
Sketch of the multifunctional disbond arrest feature (MDAF). Polymer strips carrying strain sensors are integrated into the lower adherend and paired with plain strips of the same material with equal width and thickness in the upper adherend. To simplify integration and contacting, the sensor strips are combined by a slender sideways link into an *F-shape* geometry forming a smart inlay.

The MDAF construction consists of two PVDF strips with three micro strain sensors each, pressed into the lower adherend (strap), and two PVDF strips pressed into the upper adherend (lap), as illustrated in Figure 1. While only a single pair of PVDF strips was used in the original disbond arrest feature design [37], a second pair has been added during its evolution into a sensoric concept. The two sensor rows shall enable a differential two-point measurement of the strain inside the bondline, to be correlated to its health status. Unlike the hybrid bondline concept [5,39], the PVDF inlays do not separate the epoxy adhesive. This simplifies the positioning and bonding of the CFRP parts and will be an advantage in industrial use.

In compliance with the ST method [36], PVDF copolymer (Nowofol, Nowoflon PVDF) was used as a substrate. Compared to homopolymers the copolymer foil offers excellent cold break and embrittlement properties. Thus the flexible foil is particularly suitable for aerospace applications. Most importantly, this polymer exhibits superior adhesion to the CFRP epoxy matrix after co-curing. Table 1 summarizes its mechanical properties.

For the sensing elements, a rather small strain gauge grid layout was chosen in order to adapt it to the mesh density of the prepreg material. The size of the measuring grid was dimensioned to cover several fiber crossings, so that local stress peaks and lower stress regions of the anisotropic material are averaged [40]. Due to the limited space on the 10 mm-wide PVDF strips and the desire for a high sensor density, a measuring grid length of 2.65 mm was defined. This allows multiple spatially finely resolved strain measurements along a single strip to provide information about the angle of in plane crack propagation.

**Figure 2:**
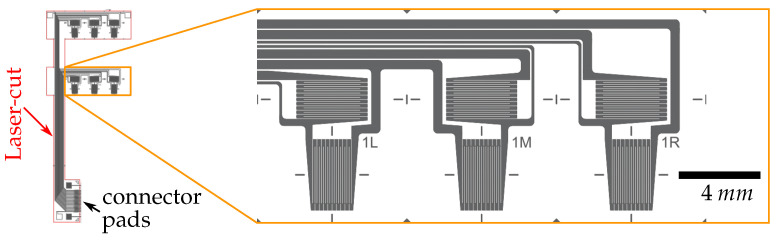
Smart inlay layout: The right part shows an enlargement of the first sensor row with the three sensor elements (1L: **left**, 1M: **middle**, 1R: **right**). Each sensor element consists of two sensor grids, one longitudinal and one with transverse orientation. The outer F-shape geometry of the inlay is created through laser cutting.

Figure 2 illustrates the chosen sensor layout. As the smart inlay is manufactured on 4 inch wafer scale its maximum size is limited. Signals from the bondline integrated sensors can be extracted by in plane conductive paths leaving the overlapping area of the bond. Thus, the two strips crack stopping feature was combined with a sideways, orthogonal link to form a single, easy to apply foil, which yielded the *F-shape* of the smart inlay. Stress peaks around the lateral PVDF strip and a non-symmetrical stress distribution inside the bondline must be accepted though. Other sensor connection techniques such as externally insulated pins piercing upright through the prepreg material were avoided, as they diminish CFRP stability [41].

Sensors on the two adjacent PVDF strips have a different distance from an emerging crack front in the finished composite specimens. In the healthy state, the entire MDAF is located well within the bondline in an area that is only slightly loaded. As the sensors are located close to each other, their outputs are similar and the difference is close to zero—even under load, as long as there is no crack. This difference starts to rise if a crack approaches the MDAF as the load of the sensors in the first row increases, while the sensors in the second row remain in the initial load state. This is because the strain peak at the crack tip decays within the distance of 10 mm between the two rows [35]. The two-row F-shape design has another advantage. If a crack is arrested at the first row of MDAF, its sensors might fail due to the stress peaks, whereas the only slightly loaded sensors in the second row remain intact. Hence, even if the sensors of the first row are damaged, the overall integrity of the adhesive layer is not jeopardized because the first row sensors log the crack initiation until their destruction while the second row sensors remain functional. If the crack passes the first PVDF barrier due to severe overloading, the output of the second row sensors can provide a “critical crack emergence signal”, even if the first row is destroyed. In addition, the second barrier also prevents the crack from rapidly growing further. For this reason, the chosen MDAF design with two adjacent PVDF inlays can provide a fail-safe functionality of both the sensor and the disbond arrest system.

A single plane half-bridge design (one sensor element contains two active orthogonal aligned measuring grids) was chosen for the sensor to compensate temperature effects. In contrast to a quarter bridge, this provides a voltage level that allows high-impedance measurements, so that the line resistance of the measurement path is of less influence. Gold was chosen as sensor material because of its low reactivity and ductility. Due to thermal and mechanical stresses in the subsequent integration process steps, other materials commonly used for strain sensors, which are often better temperature compensated, are unsuitable. The stress may result in changes in mechanical and electrical properties as well as morphology. The thickness of the golden sensor layer was adapted to accomplish a measurement grid resistance of 350 Ω. Since composites have a poor heat conductivity, the sensor resistance must be high to avoid localized heat spots within the adhesive layer.

To reduce the influence of conduction losses inside the lateral conductive tracks on the strain signal, these tracks were reinforced with electroplated copper. This also increases their mechanical strength so that signal transmission is guaranteed in the case of a propagating crack. In addition, it enables the soldering of a Surface Mount Technology (SMT) connector on the smart inlay. The short horizontal section of the tracks on the F-sensor crossbar is not electroplated to simplify sensor production. Although these short tracks are 8 times as wide as the measuring grid conductors, they noticeably contribute to the overall resistance (approx. 10% of overall resistance), which attenuates the signal. Since the tracks are orthogonal to the load direction of the specimens, its resistance change is negligible.

## 3. Materials and Methods

The sensor structures were applied and structured directly on the ductile PVDF material, using micro fabrication methods to form a flexible smart inlay.

### 3.1. Smart Inlay Fabrication

**Figure 3:**
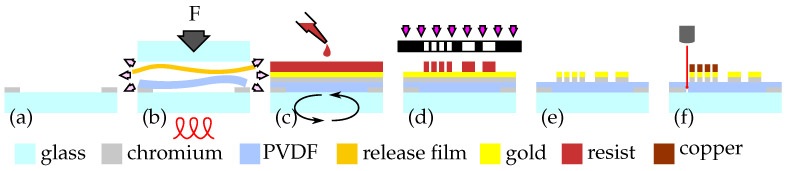
Smart inlay micro fabrication process: (**a**) Sputtering chromium ring with shadow mask. (**b**) Fixing PVDF substrate to the carrier wafer under vacuum and heat. (**c**) Sputtering the chromium gold layer and applying photoresist. (**d**) Transferring pattern by n-tone lithography. (**e**) Etching of metallic layers and removing resist. (**f**) Electroplating wires and laser cutting.

The smart inlay fabrication process is illustrated in Figure 3. The PVDF foil (Nowofol, Nowoflon PVDF) with a thickness of 100 μm was fixed onto a 4 inch glass wafer (Schott, Borofloat 33) coated with a 20 nm-thick chromium ring as adhesion promoter inside a bonding machine (Electronic Vision Co., AB-1PV), where a piston flattened the foil inside the vacuum chamber.

A temperature treatment above the melting point (see Table 1) at 210 ${}^{\circ }C$ for 1 h allowed gaseous inclusions and solvent residues to be expelled and surface damage to be eliminated. A spin rinse with acetone removed oily remains and chemically dissolved the foil surface slightly, which additionally improved the adhesion of the metallic sensor layer. The surface quality achieved was evaluated by roughness measurements with a confocal laser scanning microscope (CLSM) (Keyence, VK-X260K) in accordance with DIN EN ISO 4288. Processing continued by sputtering the thin chromium adhesion layer (approx. 10 nm) and the gold sensor layer with a thickness of 200 nm (von Ardenne Anlagentechnik, LS 440S). Structuring was achieved by lithographic means using a negative-tone photoresist and the associated developer (micro resist technology, ma-N 1420, ma-D 533/S). After wet etching of the metallic layers, the conductive tracks and soldering pads were electroplated with copper to a thickness of 7 μm.

Lastly, the F-geometry of the smart inlay was cut out using a femtosecond-laser (3DMicromac, microSTRUCT C workstation equipped with Light Conversion, Pharos-15 W). In the cutting process, electrical ridges contacting the conductive paths for the electroplating process were separated. Before the smart inlays were detached with tweezers from the carrier wafer, the sensor grids can be contacted individually with the aid of fine needles on a probe station.

### 3.2. Static Tensile Test of the Smart Inlay

**Figure 4:**
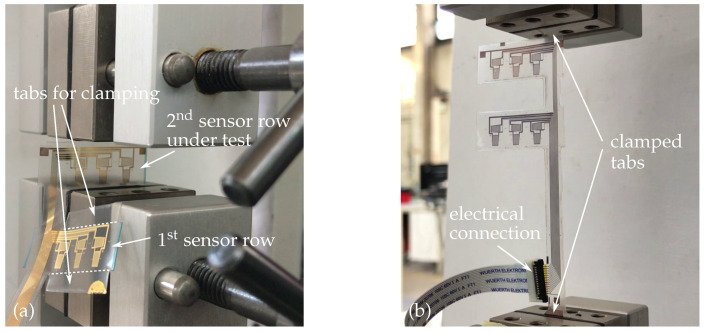
(**a**) Tensile testing of second sensor row. (**b**) Tensile testing of conductive tracks.

The F-shaped smart inlays were studied in static tensile tests. Typical ultimate design loads for mechanically fastened CFRPs in the aviation industry are defined as strains between 2500 μm $m^{-1}$ and 4000 μm $m^{-1}$ and for bonded honeycombs even as 5000 μm $m^{-1}$. The design ultimate loads are generally 50% more than the design limit loads [3]. Hence, the integrated micro strain sensors ideally withstand strains of ≈3500 μm $m^{-1}$. These requirements are demanding as commercial high-strength strain gauges on a specially equipped substrate bear strains of 2500 μm $m^{-1}$ at 1 million cycles [40] only. To determine the maximum load capacity, the smart inlays were stretched in a tensile testing machine (Zwick/Roell, Z1.0) until sensors and conductive tracks electrically fail. For this purpose, the cut out F-geometry was slightly adapted so that both sensor and conductive path strain could be investigated individually. In order to test the measuring grids under load, the foil area near the sensors was widened to provide the necessary clamp length for the jaws (see Figure 4a). Additional tabs were added above and below the vertical link of the F-shape for straining the conductive tracks only (see Figure 4b).

After mounting the smart inlays in the testing machine, the sensors were electrically connected via a ribbon cable and slowly stretched. The elongation value was set to zero once a normal force of 0 N was measured. Samples were stretched until a complete electrical failure occurs (refer to Section 4.2). A strain gauge amplifier (HBM, QuantumX MX1616B) acquired the sensor signals.

### 3.3. Fabrication of cls Specimens with Multifunctional Disbond Arrest Feature

**Figure 5:**
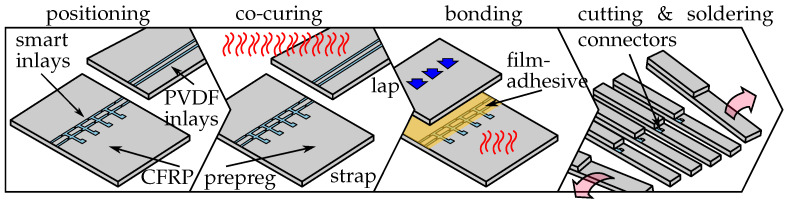
CLS specimen preparation inspired by the co-curing process developed by Löbel [39].

The manufacturing of the specimens consists of four stages (see Figure 5). After cutting and extraction from the carrier wafer, the smart inlays were placed on a wet prepreg stack (strap plate) using a positioning stencil. Plain PVDF inlays without sensors were placed on a second CFRP adherend (lap plate) in parallel. Second, the PVDF inlays were bonded to the two CFRP-adherends in a co-curing process. After cooling, the two CFRP-plates were bonded together to form a single sided overlap joint. After the curing of the adhesive, the bonded plates were cut into cracked lap shear (CLS) specimens. This type of specimen offers an unambiguous crack initiation location, as well as a defined crack growth direction, which are important for studying the disbond arrest and crack detection capabilities of the MDAF. Finally, zero insertion force (ZIF)-connectors were soldered to the connector pads.

**Figure 6:**
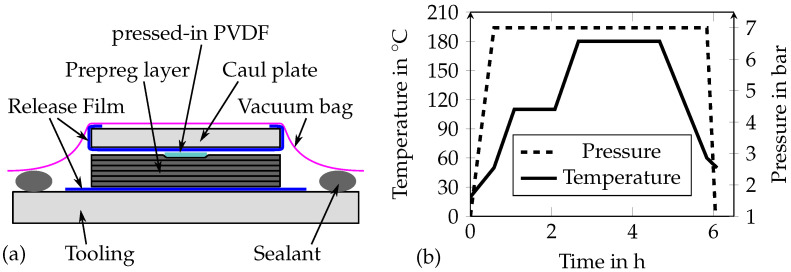
(**a**) Setup for co-curing the inlays to the CFRP adherends. (**b**) Co-curing autoclave process.

The adherends were made of 16 plies Hexply 8552/IM7 (12K) unidirectional prepreg with the stacking sequence [0/+45/90/−45/0/+45/90/−45]_s_. The 0${}^{\circ }$ outer layer minimizes the risk of delamination failures close to the bondline (see Section 4.3). The sizes of the adherends forming the strap and lap are 390 mm by 350 mm and 240 mm by 350 mm, respectively. The smart inlays were placed in a row on the CFRP strap laminate, whereas PVDF inlays without sensors were placed on the lap. Together, the PVDF strips and the smart inlay form the MDAF, as already sketched in Figure 1. The sensor structure of the smart inlay lies at the outside and is thus electrically isolated from the CFRP prepreg by the substrate. Finally, the adherends were covered with caul plates of the same size as the adherends. The caul plates ensure a smooth surface of the adherends and that the inlays are pressed into the laminate completely. Moreover, they were wrapped into a release film, which allows demolding after the curing process. The filigree sensor structures were in direct contact to the wrapped caul plate during the curing process. Both stacks were placed on a metal tooling, which was also covered by a release film and sealed between the tooling and a vacuum bag. The co-curing setup is illustrated in Figure 6a. The adherends were cured in the temperature and pressure cycle given in Figure 6b under a vacuum of less than 0.3 mbar. After co-curing, the rough sides of the CFRP plates were cut off to obtain clean edges for the subsequent adhesive bonding process.

Prior to bonding, the adherends were thoroughly cleaned with isopropyl alcohol and the surfaces were treated with an atmospheric pressure plasma using a plasma generator (Plasmatreat, FG (5001)) with a RD 1004 nozzle. A conical plasma jet head with concentric 4 mm vent, a nozzle distance of 15 mm and a row pitch of 3 mm was chosen based on previous experimental investigations [5,39]. The measuring grids were directly subjected to the plasma beam during this treatment. For the bonding process the two adherends were placed on top of each other with a layer of epoxy film adhesive (Henkel, Loctite EA 9695 0.05 PSF K) in-between. An artificial disbond was realized by placing a release foil in-between the adhesive and the strap at the first 10 mm of the overlap edge. The adherends were arranged so that their PVDF inlays align. The electrical connector pads of the smart inlays remain uncovered for later soldering. Then, the bondline was cured under vacuum in an autoclave ( 2 h at 130 ${}^{\circ }C$ and 3.0 bar). After bonding, the plate was cut into 25 mm wide CLS specimens.

**Figure 7:**
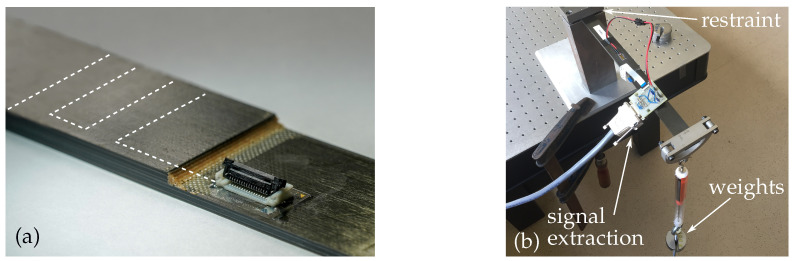
(**a**) Overlap edge of a CLS specimen with integrated mdaf; the white dotted line indicates the smart inlay in the strap. (**b**) Cantilever setup for MDAF functionality tests.

In the final step, each CLS specimen was equipped with a ZIF connector using SMT reflow soldering in an oven. Due to the comparatively low melting temperature of the PVDF substrate, a special solder paste (almit, LFM-65W A75C(L) 11%) was used. A finished CLS specimen is depicted in Figure 7a.

### 3.4. Sensor Verification

The smart inlays must endure harsh manufacturing conditions at elevated temperatures, especially during co-curing, adhesive bonding and reflow soldering of the electrical connector. Thus, after the smart inlay integration, the sensors were electrically evaluated via the ZIF connector.

For investigating the MDAF functionality, the CLS specimen was clamped horizontally on one side, thus forming a cantilever to which weights were added at the free tip (see Figure 7b). Additional 100 g weights were attached to the tip every 30 s. After 10 weights, they were gradually removed again at the same rate. The signals of both sensor rows were recorded. The longitudinal strain in the CLS cantilever grows from zero at the free end to its maximum value at the fixed end. Thus, a strain gradient occured between the two sensor rows. However, the strain is expected to be rather low in the sensor array, as it is positioned near the neutral plane of the CLS specimen.

## 4. Results

The successful integration of smart inlays is an indispensable prerequisite for the function-compliant MDAF. Therefore, special micro fabrication procedures were established to minimize the disturbance of the adhesive layer, which cannot be achieved with state of the art sensors, such as strain gauges. Moreover, the F-shape inlay is designed to allow the detection of stress gradients inside the bondline, which are characteristic of propagating cracks.

### 4.1. Evaluation of Smart Inlay Fabrication

In first sensor fabrication experiments, sensors were structured on the PVDF foil without wafer fixation and surface pretreatment. The rough, untreated surface caused electrical disconnection in the sensor grid, even for rather small elongations, and thermal stresses during the composite integration caused many failures.

**Figure 8:**
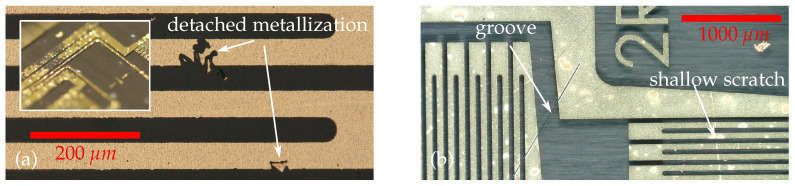
(**a**) Micrograph of an interrupted measuring grid after lithografic structuring on the untreated foil substrate. Insert shows picture of larger detached metallization flakes. (**b**) Micrograph obtained with ring light illumination allows to distinguish between function disturbing grooves and superficial scratches.

Figure 8a shows detached metallization due to oily residues that were found on the untreated PVDF foil, which probably originated from the roll-to-roll manufacturing at the vendor. Simple isopropanol foil cleaning did not remove these. Microscopic examinations also revealed interruptions of the filigree measuring grids (where conductive measuring tracks have a width of only 50 μm to achieve the nominal sensor resistance) due to surface defects such as grooves and ridges in the PVDF foil and small craters in the proximity of solvent inclusions (see Figure 8b). Cleaning the film with strong solvents such as acetone has initially been avoided since ketones have a dissolving effect on PVDF. However, we discovered that removing impurities using solvent spin rinse was not harmful to the integrity of the foil. Instead, the slightly dissolved surface positively supports the adhesion strength of the subsequently sputtered thin film.

**Figure 9:**
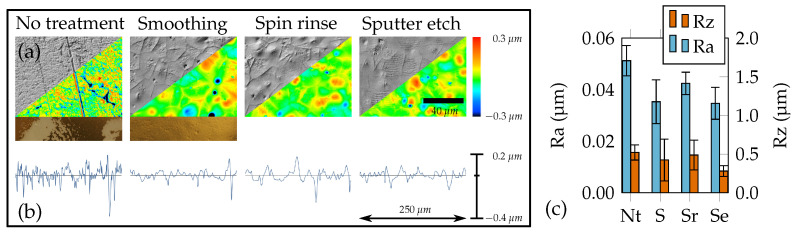
PVDF surface as obtained with various substrate pretreatments. (**a**) Four CLSM images, upper left triangle: Differential interference contrast images, lower right triangle: topography scans. The two supplementary images under “no treatment” and “smoothing” show tape peeling tests of a substrate coated with gold. (**b**) Roughness line scans (with identical scaling). (**c**) Effect of the surface treatments steps on roughness parameters “Ra” and “Rz” with standard deviation.

The tape peeling test as visualized in Figure 9a showed, that the adhesion after the introduction of the wafer fixation and smoothing process was already sufficient to completely prevent detachment between metallization and the fully covered substrate. Thus, the influence of localized spots of contamination with limited adhesion becomes visible only after the structuring of the filigree functional structures. This substantial improvement in substrate quality also became visible with respect to surface roughness (see Figure 9b,c). The PVDF surface with no treatment (Nt) has a fine grain structure with some rather deep grooves such that “Ra” value was the highest. After melt down during the smoothing (S) process, the surface exhibits a plain orange skin-like appearance, with some small craters where solvents have evaporated. Hence, the “Ra” value drops. After the aceton has slightly dissolved the surface in a spin rinse (Sr), grain boundaries seem to stick out more whereas craters have flattened. Sputter etching (Se) flattens the relief-like elevations such that the “Rz” value finally decreases to a value significantly smaller than for the untreated foil. In addition, the rigid carrier wafer enables simplified handling and improved micro fabrication, e.g., with regard to photoresist layer thickness homogeneity.

In spite of the improvements made preparing the foil surface, sporadic and randomly located detachments between thin film structures and the substrate remained. However, adhesion of the sputtered layer was additionally improved by adjusting the sputtering power. Higher-power coatings ( 200 W) were beneficial for adhesion but led to high intrinsic layer stresses as well. The latter created a wavy substrate and made the structures more brittle under load. We found a sputtering power of 100 W to be a good compromise between proper adhesion and low layer stress.

### 4.2. Smart Inlay Robustness

**Figure 10:**
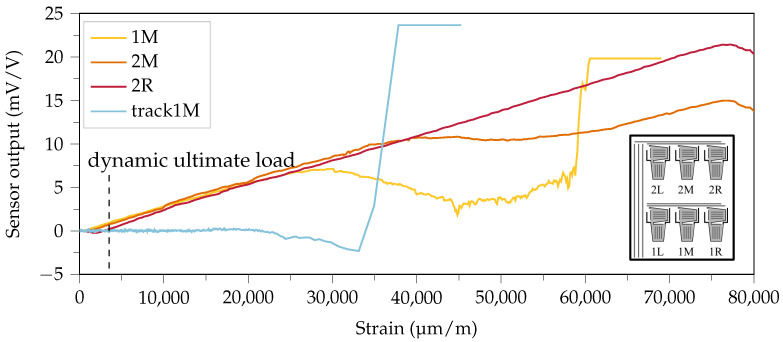
Exemplary sensor signals obtained under tensile load: First row middle sensor element (1M) was the first to deviate from the desired linear elastic characteristic. The middle element in the second row (2M) started decaying at 40,000 μm $m^{-1}$. The last of the six sensor elements to loose function was the right element in row two (2R). With a slightly different sample (refer to Section 3.2), the vertical conductive tracks where lengthened, while the sensor signal from sensor element 1M was logged.

Figure 10 shows exemplary sensor signals of the smart inlay obtained during the static tensile tests described in Section 3.2. In the first tests, the sensors have been stretched until complete failure. The initial functional degradation occurs for sensor 1M at a strain of 25,000 μm $m^{-1}$. The middle sensor in row two (2M) shows the desired linear elastic behavior up to a strain of 40,000 μm $m^{-1}$, while sensor 2R yields the largest maximum strain of more than 75,000 μm $m^{-1}$. Thus, in this one cycle static test typical aviation, ultimate design loads stated in Section 3.2 were exceeded. Note that these ultimate loads refer to dynamic loading which typically causes faster material degradation. In addition, the signal transmission must also function at very large elongations. Due to the F-design, the conductive tracks are located directly in the area of the progressing crack front and must therefore be very robust and insensitive to strain.

To verify this, they were stretched in the tensile testing machine without straining the measuring grids. The measurement at sensor element 1M shows a stable zero signal up to an elongation of approx. 20,000 μm $m^{-1}$ and thereby confirms the robustness of the conductive tracks.

### 4.3. Evaluation of Sensor Integration and cls Specimen Fabrication

**Figure 11:**
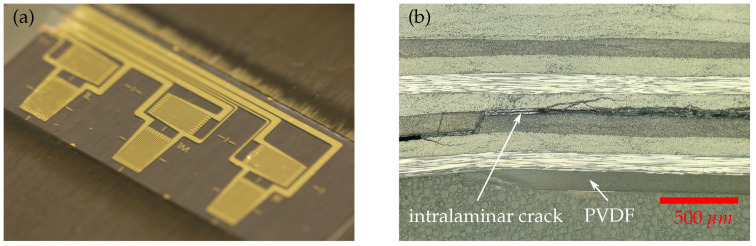
(**a**) Thin-film sensors co-cured to a CFRP-adherend. (**b**) Cross section sample of ruptured cls strap with interlaminar crack propagation. Sample was gained from a cls specimen with integrated MDAF that was pulled until a complete failure of the bondline was achieved.

The function conformity of the MDAF with respect to adhesion, crack arrest and crack detection was investigated using CLS specimens. The smart inlays integrate well (see Figure 11a) and withstand the harsh co-curing conditions. The process works reliably with standard tools. Successful co-curing must ensure that the sensor shape is preserved and the adhesion between the CFRP plates and all inlays is better than the adhesion between the adhesive and the CFRP matrix. If any interface of the MDAF exhibits poorer adhesion than the bondline, this would be a preferred location for crack initiation and therefore counteract the desired functionality. Figure 11b illustrates the results from a preliminary adhesion test, where a CLS specimen was loaded till a complete failure of the bondline occured. The cross sectional micrograph of this sample shows, that the CFRP matrix material ruptured intralaminarily before the interface between PVDF inlay and CFRP failed. This is clear evidence for outstanding adhesion strength between the inlay and the surrounding matrix material.

**Figure 12:**
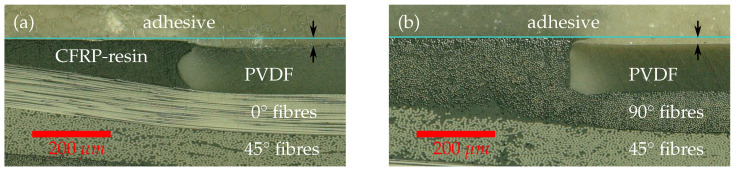
Cross section view of CFRP layers with polymer inlay. A light blue line is introduced to indicate the surface level of the CFRP material. Although the PVDF inlay was pressed flush with the Caul plate into the prepreg, a shrinkage step of about 25 μm, marked by arrows, occurs when the cured CFRP plate cools down due to the different coefficients of thermal expansion. (**a**) Upper layer orientation is 0°. (**b**) Upper layer orientation is 90°.

The upper ply with 0${}^{\circ }$ orientation in Figure 12a yields the maximum strength at the PVDF interface in the direction of longitudinal strain whereas the strength of the 90${}^{\circ }$ ply in Figure 12b under the same longitudinal load is much lower. As a result, damages in the 90${}^{\circ }$ ply can be initiated much easier and at loads below the strength of the adhesive. Hence, the fracture mechanism is likely dominated by delaminations if a 90${}^{\circ }$ ply is used at the interface instead of a cohesive failures in the adhesive. For that reason, 0${}^{\circ }$ plys at the interface to the crack stopping polymer stripe are recommended [3,42]. In addition, the cross sectional view revealed a shrinkage step of about 25 μm towards the PVDF inlay, caused by different coefficients of thermal expansion between the CFRP epoxy matrix and the PVDF material.

An optical inspection after the plasma treatment prior to adhesive bonding does not show any damages to the sensor structures. No direct optical inspections were possible to check for the sensor integrity after the adhesive bonding process was completed. There was no evidence that the bonding process or reflow soldering of the ZIF connector causes damage to the sensors systematically.

### 4.4. Sensor Integration Yield

**Figure 13:**
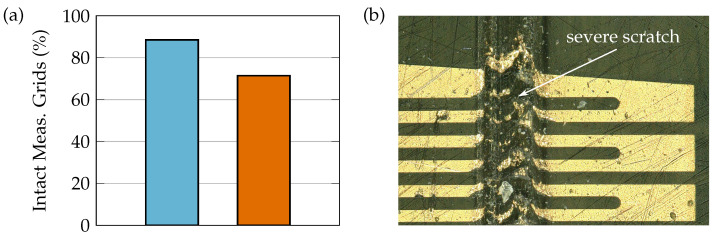
(**a**) Percentage of electrically intact measuring grids. About 90% of working measuring grids were obtained directly after micro fabrication on the carrier wafer (blue bar). After CFRP integration, approx. 70% of the sensor structures are intact (orange bar). (**b**) Damaged integrated sensor structure due to handling of co-cured plates on rough surface.

In a research environment, the high yields of industrial production can not be met, but trends for critical process steps can be identified. The smart inlays were investigated for proper functionality prior to and after CFRP integration, by determination of failure rates of individual measuring grids by resistance measurements. Achieving the desired nominal resistance is the criterion for an intact measuring grid. However, this does not allow one to conclude that the sensor will also function permanently under dynamic loading. For example, an electrically intact measuring grid can fail due to mechanical weak spots of the substrate such as scratches within a few load cycles, e.g., if the structured metallic thin film detaches from the substrate under shear stress. For the statistics shown in Figure 13a, a total of 360 sensor grids (30 manufactured smart inlays with 12 sensor grids each) on PVDF substrate have been evaluated.

On average, 90% of the measuring grids on a smart inlay was electrically intact after production in the clean room and separation of the sensors by means of femtosecond lasers. After CFRP integration of these inlays, this value drops further by 20%. The thermal and mechanical stress in the autoclave process acts here like artificial aging. Thus, electrically intact grids which were slightly damaged during production, additionally fail. Nevertheless, the vast majority of the of the smart inlay sensor structures survive the integration process. At the most critical moment, when the PVDF substrate is melted in the co-curing process and starts floating, the filigree sensor structure must be stabilized. It was found that the mechanical contact with the wrapped caul plate was essential and allows the integration in a standard curing process. But for curing set-ups in which the substrate of the sensor is melted while its structure is not stabilized the integration is likely to fail.

Before adhesive bonding, caution is required for handling the co-cured plates (see Figure 13b). A simple solution was found by covering the integrated sensors in this phase by means of a slightly adhering foil which was removed just before adhesive bonding. Following this method there was no additional increase in the sensor fail rate due to plasma pretreatment and the second autoclave cycle for the adhesive bonding.

### 4.5. Verification of Differential Measurement Principle

**Figure 14:**
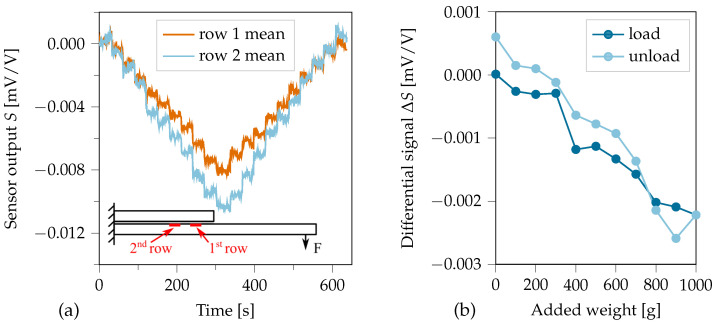
Signals observed in cantilever testing. (**a**) Sensor signals over time, while 100 g weights were added to and removed from the cantilever tip with 30 s intervals. The little insert shows a side-view sketch of the specimen indicating the positions of the acting force and of row one and two sensors. (**b**) The differential sensor signal $\Delta S=S_{row2}-S_{row1}$.

The cantilever test illustrates the strain gradient detection with the differential measurement principle with the two sensor rows of the F-shaped smart inlay. Assuming a homogeneous adhesive layer thickness of 100 μm and that lap and strap are built symmetrical to the exact same height, the distance between the sensors and the neutral plane is only 50 μm. To observe a significant delta in signal amplitudes between the two sensor rows therefore requires a highly sensitive measuring system. The mean sensor values for row one and two in Figure 14 on the left show, that the levels of additionally added weights are clearly visible. However, the steps do not show an absolutely stable plateau over the 30 s period. Both signals carry a negative sign which is expected as the sensors are located in the lower part of the bent specimen, which was under compression. Most importantly, a clear difference between the signal amplitudes of first and second row sensors can be seen. As the bending moment increases towards the restraint, the sensors of row 2 located closer to the restraint, were subjected to a higher strain, which is in good correlation with the measuring data. From the differential signal plot it can be seen that a small offset remains as hysteretic effect after complete unloading of the specimen. As the distance between sensor rows can be assumed constant, differential and gradient signal are proportional to each other.

Even though bending the specimens is not associated with crack propagation, the load-dependent differential signal was obtained representing a stress gradient. For real crack propagation, the highest strains in the bondline occur at the crack front. Thus, a strain gradient will be derivable from the difference between the measured values of the front and rear sensors. The measurable strain gradient under longitudinal strain causing crack propagation will be several magnitudes larger though.

## 5. Discussion & Outlook

A first novelty of our work lies in the successful implementation of a thin-film sensor array on a PVDF substrate, a material whose crack stopping capability is confirmed. We were able to show how PVDF foil must be pretreated to be suitable as a substrate for lithographic micro fabrication. Most importantly, the surface of the foil has to be smoothed in order to avoid electrical disconnection in the sensor grid. A second novelty lies in the successful CFRP integration of the smart inlay. The presented integration technology is not restricted to PVDF substrates, but is also likely to function with other plastics showing good adhesion to composite materials [17,43]. Even though the sensor survival rate can still be improved, our investigations demonstrate a stable integration process for thin film sensor embedding. Here, we found that the mechanical contact between the smart inlay and the wrapped caul plate stabilized the filigree thin film structures, such that they remained functional even though the substrate was completely melted during CFRP integration. The smart inlays build the core of a novel MDAF for bonded composites.

Three industrial application scenarios for the hybrid bondline in aircraft structures were proposed [39]. These are double joints for stiffeners, wide overlap joints between fuselage sections or fuselage barrels, and sandwich plates. For all of these, multiple disbond arrest features are required for a sufficient damage size limitation. The proof that the smart PVDF inlays can be integrated by standard manufacturing processes, demonstrates the potential for the integration of MDAFs in industrial processes. This can be done by simply placing the smart inlays on the wet prepreg before co-curing. Compared to the hybrid bondline [5], the concept requires less positioning accuracy of the inlays and the bonding process can be achieved with a continuous adhesive layer without interruptions or intermediate steps because no interlayers of the thermoplastic material are required. With lithografic structuring, the sensor density can in the future be increased to a high level if necessary, so that cracks can be spatially localized very precisely. In the long-term, rolls with thermoplastic foils carrying the micro sensor arrays can be prepared. The placement of the smart inlays is then possible in a single step by a conventional automated tape laying process. Larger format inlays require an up-scaled fabrication, for instance roll-to-roll processes. High temperature laminating devices and the cost efficient use of screen printing technology could be a valuable option. Their potential will be evaluated in the future.

The in-plane orthogonal sensor design simplifies the structuring of the sensors and is insensitive to temperature changes nonetheless. However, the design is only suitable to a limited extent for strain analysis, since the influences of longitudinal and transverse strain are mixed [44]. In this study, the special structure of the CLS specimen ensures a defined crack starting point and direction of propagation. The load case is similar to the one in doubler joints for stiffeners. The CLS specimens thus not only enable studying the multifunctional bondline, but also reflect a MDAF for a typical load case in modern aircrafts. For the other two aforementioned application scenarios, however, other investigations like tests with wide single lap shear specimens or sandwich structures are necessary in order to adjust the MDAF design to these load cases.

The results from the quasi-static tensile test of the smart inlays demonstrate that the foil sensors withstand a static load of 20,000 μm $m^{-1}$, which is considerably more than the limit load elongation level of ≈3500 μm $m^{-1}$ under dynamic load, which is a standard specification for aircraft construction. However, further studies are necessary to prove the long-time stability and the crack monitoring capability of the smart inlay, when it is integrated into the composite as MDAF and loaded dynamically.

Finally, the capability of the MDAF for a differential strain measurement was demonstrated in a cantilever test. Hence, the approach to monitor the bondline integrity by a two-point strain difference measurement can be investigated with the proposed MDAF in consecutive studies. The low bending test loads did not cause any cracks in the bonded specimen while a differential signal was measured though. Bonded overlap joints require a design that avoids any bending moments because these induce peel stresses which are the greatest threat to adhesive bonds [45,46]. Since the amplitude of a measured signal caused by bending is orders of magnitudes smaller than that due to crack propagation, the latter will always be clearly distinguishable. This has already been shown by the experimental studies on CLS specimens of the hybrid bondline. Here, small bending moments are induced in CLS specimens as well, due to the eccentric load. These bending moments cause a small strain gradient between the sensor positions in the undamaged probe whereas a much higher difference occurs if a crack approaches the MDAF [35]. In addition, the reliability of crack detection can further be improved with more sophisticated evaluation algorithms. A thorough investigation of the proposed damage criterion must still be developed and the respective loading conditions for different application scenarios must be taken into account.

The crack arrest capability of the multifunctional bondline, the initial mechanical strength, as well as the residual strength after crack arrest require extensive investigations. Hence, these properties are obtained in quasi-static and cyclic tests in the future. The study at hand provides the design and manufacturing process of the CLS specimens containing a MDAF for these consecutive investigations.

## Figures and Tables

**Table 1:** PVDF Properties.

Material Property	Nowoflon 100 µm
Young’s modulus [MPa]	1000
Yield stress [MPa]	40
Yield strain [%]	10
Poisson’s ratio	0.46
Melting point [°C]	163

## Data Availability

The raw data of the experiments can be requested from the authors.

## Abbreviations

The following abbreviations are used in this manuscript:

CFRP	carbon fiber reinforced plastic
CLS	cracked lap shear
CLSM	confocal laser scanning microscope
FBG	fiber Bragg grating
FRP	fiber reinforced plastic
MDAF	multifunctional disbond arrest feature
PI	polyimide
PVDF	polyvinyliden fluoride
SHM	structural health monitoring
SMT	Surface Mount Technology
ST	surface toughening
ZIF	zero insertion force
